# Complex association between rural/urban residence, household wealth and women’s overweight: evidence from 30 cross-sectional national household surveys in Africa

**DOI:** 10.1186/s40608-016-0141-1

**Published:** 2017-01-19

**Authors:** Nyovani Janet Madise, Gobopamang Letamo

**Affiliations:** 1 Division of Social Statistics and Demography, University of Southampton, SO17 1BJ Southampton, UK; 2 Department of Population Studies, University of Botswana, Block 242B Room 011, Private Bag UB 00705 Gaborone, Botswana

**Keywords:** Sub-Saharan Africa, Overweight, Obesity, Urban/rural residence, Wealth, multilevel

## Abstract

**Background:**

We sought to demonstrate that the relationship between urban or rural residence and overweight status among women in Sub-Saharan Africa is complex and confounded by wealth status.

**Methods:**

We applied multilevel logistic regression to data from 30 sub-Saharan African countries which were collected between 2006 and 2012 to examine the association between women’s overweight status (body mass index ≥ 25) and household wealth, rural or urban place of residence, and their interaction. Macro-level statistics from United Nations agencies were used as contextual variables to assess the link between progress in globalization and patterns of overweight.

**Results:**

Household wealth was associated with increased odds of being overweight in nearly all of the countries. Urban/rural living and household wealth had a complex association with women’s overweight status, shown by 3 patterns. In one group of countries, characterised by low national wealth (median per capita gross national income (GNI) = $660 in 2012) and lower overall prevalence of female overweight (median = 24 per cent in 2010), high household wealth and urban living had independent associations with increased risks of being overweight. In the second group of less poor countries (median per capita GNI = $870) and higher national levels of female overweight (median = 29), there was a cross-over association where rural women had lower risks of overweight than urban women at lower levels of household wealth, but in wealthier households, rural women had higher risks of overweight than urban women. In the final group of countries, household wealth was an important predictor of overweight status, but the association between urban or rural place of residence and overweight status was not statistically significant. The median per capita GNI for this third group was $800 and national prevalence of female overweight was high (median = 32% in 2010).

**Conclusions:**

As nations develop and household wealth increases, rural African women are at increased or higher risk of being overweight compared with urban women. Programmes and policies to address rising prevalence of overweight are needed in both rural and urban areas to avoid serious epidemics of non-communicable diseases.

## Background

Sub-Saharan Africa (SSA), like many other regions of the world, is experiencing an increase in overweight and obesity even though the region is still grappling with the persistence of under-nutrition which is implicated in many of child deaths annually [1]. According to the World Health Organization (WHO) Global Health Observatory, approximately 35% of SSA adult women are either overweight or obese, not far from the global estimate of 40% [2]. The main determinants of this increase in the prevalence of overweight are thought to be globalization, increase in wealth and urbanization which operate through changes in food supply systems, changes to diets and physical inactivity [3–7]. The health and economic implications of being overweight or obese are serious since this is a risk factor for many non-communicable diseases (NCDs) including cardiovascular diseases, hypertension, arthritis, cancers and diabetes mellitus [8, 9]. Estimates of NCD-related mortality in Africa indicate that there were approximately 2.1 million deaths in 2010, up by 46% from 1990 [10].

The availability of nationally representative anthropometric data has highlighted the scale of overweight status and associated NCDs in SSA. The southern African countries including South Africa, Botswana, Namibia, Lesotho and Swaziland, have some of the highest prevalence of female overweight in SSA. Surveys conducted between 2007 and 2013 found that in Botswana, approximately 53% of women 25–64 years old had body mass index (BMI) ≥ 25 and 37% had raised blood pressure [11, 12]. In Lesotho, the prevalence of overweight among women was 58 and 36% had raised blood pressure [13] and in Swaziland, more than 65% of women were overweight and 35% had raised blood pressure [14]. Even in countries such as Malawi where about 50% of under-five children are stunted, close to 30% of women are overweight [15]. High prevalence of overweight among females could have long-term implications of inter-generational transfer of NCDs, giving rise to future generations with a pre-disposition to overweight and NCDs [16, 17].

The transition to overweight status in SSA is truly underway and urbanization is generally considered to be the main driver. While much research has been conducted on overweight status in urban areas of low and middle income countries (LMIC) [18–20], there is a gap in knowledge on the scale of overweight status in rural areas. The limited evidence that exists, although not generalizable, suggests that overweight status is increasing in rural areas also and this phenomenon is happening at quite low levels of household wealth. Keding et al. found that in rural Tanzania, there were three times as many overweight or obese women than thin women in the communities [21]. Similarly, Kirunda et al.’s study in peri-urban and rural areas of eastern Uganda found quite high levels of overweight and obesity among rural men and women, roughly around 20%. Indeed, Popkin et al. have reported of higher relative annual change in obesity levels in rural areas compared to urban areas in parts of Africa [22].

**Fig. 1:**
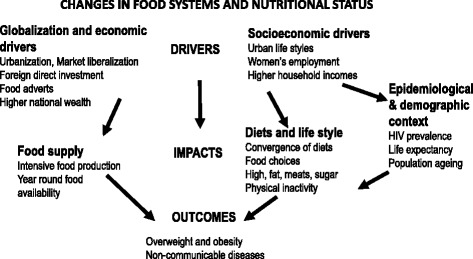
Conceptual framework for studying the impact of globalization on nutritional status. Adapted from Kennedy, Nantel and Shetty (2004)

The arguments put forward for the increase in the prevalence of overweight in both urban and rural areas are well captured in the conceptual framework proposed by Kennedy et al. [7] for understanding the drivers and impacts of globalization on food systems and nutritional status (see Fig. 1). Globalization is thought to alter rural food production systems from subsistence to intensive agriculture and to encourage the influx of cheaper processed foods onto the food market as a result of market liberalization and foreign direct investment [6, 7]. Socioeconomic drivers such as urban life styles (urbanism), food advertisements, women’s participation in the labour force, and household wealth influence physical inactivity and dietary preferences. The consequences of these changes are an increase in the prevalence of overweight, and non-communicable diseases. In Fig. 1, we have modified Kennedy at al’s framework as follows: inclusion of urban life style (urbanism) to replace rural-to-urban migration among socioeconomic drivers since adoption of urbanism can occur in rural areas; classification of ‘sedentary lifestyles’ as an ‘impact’ of socioeconomic drivers and not as a driver of changing diets as suggested in the original framework; and the addition of the epidemiological and demographic context to reflect arguments from the epidemiological transition that as countries develop, the disease profile changes to higher burden of NCDs, life expectancy increases, and the population ages as fertility drops [23]. We have also added HIV prevalence because where this is high, there have been reports of preferences of body shapes towards overweight or obese status [24–26].

In this paper, we make a unique contribution by examining the complex relationship between household wealth, urban or rural living, and overweight status in sub-Saharan African countries. In particular, we examine how this association changes as countries progress with globalization and urbanization. Drawing from the literature, we make the following hypotheses:

1. Household socioeconomic status is strongly associated with the risk of being overweight;
2. The association between urban/rural residence and overweight is complex and depends on wealth status;
3. As countries become more globalized and urbanized, the association between overweight status and the place where a woman lives (urban or rural) becomes weaker.

## Methods

We used data from the Demographic Health Survey (DHS) programme from 30 sub-Saharan African countries collected between 2006 and 2012 and extracted information on women’s anthropometric measurements and background demographic and socio-economic variables. Botswana did not participate in the DHS programme during this period so we used comparable nationally representative data from the 2007 Botswana Family Health Survey (BFHS). In total, data from more than 208,650 women were used. The DHS programme has, since the 1980s, conducted periodic nationally representative household surveys in low and middle income countries. The DHS are comparable over space and time although additional modules can be introduced at different phases of the programme. DHS use similar procedures for survey design and labelling across countries, making the data very popular for cross-country comparison. The response rates in DHS typically exceed 90% and field workers are trained to ensure that they take and record measurements accurately. Similar strategies for data collection are adopted for the BFHS. Therefore, we are confident that the quality of data used in this paper is good.

### Measures

#### The dependent variable

We used Body Mass Index (BMI) as a measure of nutritional status. BMI is calculated as a ratio of weight in kilogrammes and the square of height in metres. A binary dependent variable (overweight versus not overweight) was created using the WHO classification of “overweight” as BMI ≥25.00 [27]. There have been debates in the literature about BMI as a measure of overweight because it does not distinguish between body fat and lean body mass and so may be inappropriate for international comparisons since some ethnic groups have more body fat at the same BMI level [28, 29]. Proponents of BMI point out to its simplicity and the fact that it is easy to collect heights and weights in household surveys [30]. We included women aged between 15 and 49 years since the majority of DHS collect anthropometric measurements from this group and under-five children only. In calculating BMI, we excluded women who were pregnant at the time of the survey and those who had given birth in the four months before the survey since their weight measurements are affected by their pregnancy or post-partum state.

#### Independent variables

To test our hypotheses, we used household wealth, rural or urban residence, and the interaction of these 2 variables, thus enabling comparison between socioeconomic sub-groups within urban, and within rural areas. In the conceptual framework above, these variables fall under the ‘socioeconomic drivers’ category. Household wealth was a count of the number of assets and modern amenities owned by the household. The maximum number of assets was 15, but not all countries had cases up to this level. At the right-hand tail of this variable, we grouped together levels of wealth where there were too few cases (<20), thus not all countries had 15 levels of wealth status. These assets were: bicycle, car, working radio, working television, telephone, piped water, refrigerator, sole use of toilet facility, types of material for flooring, walls, and roofing, and farm equipment (Botswana only). The majority of studies in the literature use wealth quintiles computed using principal component analysis of data on ownership of assets and amenities. However, these measures of wealth quintiles are heavily biased towards assets found in towns and cities and thus they fail to distinguish between rural residents of different socioeconomic status [31, 32]. Also, where the aim is to compare levels of wealth instead of relative wealth, Garenne has proposed a simple measure of absolute wealth which is just the count of amenities and assets in a household [33]. This is the measure that we have used.

#### Macro-level variables

As indicators of globalization and urbanization, we used log base 10 of Gross National Income (GNI) per capita (using the Atlas method, current US$) and urbanization rates. As indicators of the epidemiological and demographic context we included life expectancy at birth, total fertility, and prevalence of overweight among women. The macro-level variables were extracted from the World Bank database except the prevalence of overweight which came from the WHO Global Health Observatory. They correspond roughly to the period between 2008 and 2012.

#### Control variables

Other variables under the group of “socioeconomic and demographic drivers” suggested by the literature include: age (as a continuous variable and age-squared because of a non-linear relationship with the risk of overweight), marital status, breastfeeding status, women’s highest level of educational, and contraceptive use, all of which are linked to women’s employment outside the home, religion (country specific) and region of residence (country specific).

### Statistical analysis

To test the first 2 hypotheses, we fitted logistic regression models for the odds of being overweight for each of the 30 countries with household wealth, urban/rural residence, and the interaction of the 2 variables as the key independent variables. Since most DHS use cluster sampling, (the clusters are often census enumeration areas), we used two-level random effects models where the first level was the woman and the second level was the cluster. Clustering can also be of substantive interest since individuals within a cluster may have similar determinants and risks of overweight, for example shared socioeconomic factors, similar preferences for certain foods and levels of physical inactivity. We also accounted for stratification and unequal sampling weights using the *svy* commands in STATA. Most DHS oversample urban households so that weights are required to obtain national-level estimates [34]. Failure to account for these survey design features can lead to narrower confidence intervals than is the case [35, 36].

To test the third hypothesis, we pooled the data from the 30 countries and after adjusting for the individual level variables, we added the macro-level indicators of globalization and urbanization to see if they attenuated the association between urban/rural residence and the odds of being overweight. For this ‘all Africa’ model we used a three-level random effects analysis using MLwin software, where the three levels were: country, cluster, and the individual woman.

The general two-level and three-level random effects logistic regression models can be expressed, respectively, as

$$ \begin{array}{l} log\left(\frac{P_{ij}}{1-{P}_{ij}}\right)={\beta}_1{X}_1+\cdots {\beta}_m{X}_m+{\gamma}_1{Z}_1+\cdots +{\gamma}_r{Z}_r+uj\\ {} log\left(\frac{P_{ijk}}{1-{P}_{ijk}}\right)={\beta}_1{X}_1+\cdots {\beta}_m{X}_m+{\gamma}_1{Z}_1+\cdots +{\gamma}_r{Z}_r+{\varphi}_1{H}_1+\cdots +{\varphi}_s{H}_s+{v}_k+{u}_{jk}\end{array} $$

Where subscripts *i, j, k* represent the individual, cluster, and country level, respectively; X, Z, H are covariates at the individual, cluster, and country level and *β*,$\gamma$, $\varphi$ are the corresponding coefficients; *v* and *μ* represent the between-country and between-cluster random components which are assumed to have Normal distributions with mean equal to zero and variance equal to *σ*_*v*_^2^ and *σ*_*μ*_^2^, respectively [36]. It should be noted that there were no variables in the datasets measured at cluster-level but the level was kept to preserve the hierarchy in the data.

## Results

Table 1 presents descriptive information for the 30 countries including the sample sizes of the DHS datasets that were used. Based on the averages of the indicators, the 30 countries appear to be representative of SSA. Slight differences were noted for mean GNI per capita in 2012 ($1627 for the sample compared to $1606 for SSA); life expectancy (mean difference of 2 years between the sample and SSA); and 2% difference in the national prevalence of female overweight between the mean of our sample and the SSA average.

**Table 1:** Survey information and selected characteristics for 30 sub-Saharan African countries

DHS Survey	Sample size	Per capita Gross National Income, 2012	Life expectancy at birth, 2012	% urban population in 2012	% women with BMI ≥ 25 2014	Total fertility rate	% adults HIV positive 2012
Benin 2011	13633	760	59	46	34	4.9	1
Botswana 2007	4904	7650	62	62	53	2.7	23
Burkina Faso 2010	6996	670	58	27	26	6	1
Burundi 2010	3807	240	56	11	19	6	1
Cameroon 2011	6644	1190	57	53	37	5	5
Congo Brazzaville 2011/2	4634	2550	59	64	36	5	3
Cote d'Ivoire 2012	3966	1220	53	52	34	5	3
DR Congo 2007	3800	370	52	35	24	6	1
Ethiopia 2011	13675	410	64	17	23	5	1
Gabon 2012	4599	10020	63	86	45	4	4
Ghana 2008	4185	1580	62	53	40	4	1
Guinea 2012	3876	440	58	36	29	5	2
Kenya 2008/9	7187	870	61	24	30	5	6
Lesotho 2009	3593	1480	50	28	45	3	23
Liberia 2007	5757	370	62	48	30	5	1
Madagascar 2009	7153	420	64	33	27	5	1
Malawi 2010	6405	320	59	16	27	6	11
Mali 2006	11304	660	57	36	27	7	1
Mozambique 2011	11318	510	53	31	27	6	11
Namibia 2007	8541	5700	67	39	50	4	13
Niger 2012	3889	390	59	18	23	8	1
Nigeria 2008	26486	2460	54	50	37	6	3
Rwanda 2011	6178	600	65	19	24	5	3
Senegal 2010/1	4820	1030	64	43	34	5	1
Sierra Leone 2008	2960	530	46	40	30	5	2
Swaziland 2008	4371	3100	54	21	49	4	27
Tanzania 2010	8426	570	61	27	30	5	5
Uganda 2011	2201	480	57	16	26	6	7
Zambia 2007	5775	1410	57	40	33	6	13
Zimbabwe 2010	7567	800	58	39	38	4	15
*Sample average*		*1627*	*58*	*37*	*33*	*5*	*6*
Sub-Saharan Africa		1606	56	37	35	5	5

Sources: World Bank: *GNI*, Population Size, % urban, Life expectancy, *WHO* % women with BMI > =25, *DHS* Total fertility rate, *UNAIDS* HIV rate

**Fig. 2:**
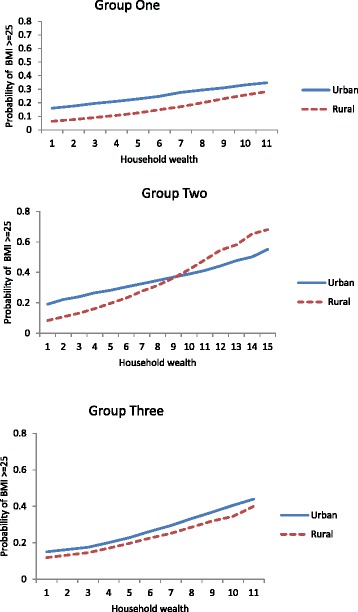
Estimated probabilities of being overweight by urban/rural residence and household wealth for 3 groups of sub-Saharan African countries

The results of the first stage analysis are shown in Tables 2, 3, 4, 5 and they support the first hypothesis that household wealth is associated with the odds of being overweight. In 28 countries this association is statistically significant at 5% level and in the remaining 2, the association is of marginal statistical significance. The regression models included the control variables (age, breastfeeding status, highest educational level, marital status, contraceptive use, religion, and region of residence). The association between the odds of being overweight and the interaction effect of household wealth and urban/rural residence is statistically significant in some countries but not others, thus partially supporting the second hypothesis. The association between overweight status and household wealth, urban/rural residence, and their interaction can be summarised by 3 main patterns which are graphically illustrated in Fig. 2.

**Table 2:** Adjusted Odds ratios of being overweight by selected characteristics

	Benin 2011 (*N* = 13633)		Botswana 2007 (*N* = 4904)		Burkina Faso 2010 (*N* = 6996)	
Characteristic	Odds ratios	95% CI	Odds ratios	95% CI	Odds ratios	95% CI
Age	1.279	(1.218, 1.343)	1.187	(1.122, 1.254)	1.152	(1.062, 1.251)
Age-squared	0.997	(0.996, 0.999)	0.998	(0.998, 0.999)	0.998	(0.997, 1.00)
Urban	1.303	(0.962, 1.764)	1.962	(1.299, 2.964)	2.983	(1.620, 5.491)
Wealth	1.110	(1.060, 1.163)	1.139	(1.095, 1.185)	1.197	(1.104, 1.300)
Wealth*Urban/rural	1.056	(0.999, 1.117)	0.942	(0.901, 0.985)	0.960	(0.867, 1.061)
	Burundi 2010 (*N* = 3807)		Cameroon 2011 (*N*﻿ = 6644)		Congo Braz. 2011/12 (*N* = 4634)	
Characteristic	Odds ratios	95% CI	Odds ratios	95% CI	Odds ratios	95% CI
Age	1.113	(1.000, 1.238)	1.209	(1.149, 1.272)	1.485	(1.334, 1.652)
Age-squared	0.998	(0.996, 0.999)	0.998	(0.997, 0.999)	0.995	(0.993, 0.997)
Urban	6.147	(2.865, 13.188)	1.945	(1.351, 2.800)	1.892	(1.042, 3.435)
Wealth	1.242	(1.090, 1.416)	1.111	(1.052, 1.174)	1.177	(1.093, 1.268)
Wealth*Urban/rural	0.864	(0.749, 1.000)	0.944	(0.882, 1.010)	0.938	(0.823, 1.032)
	Cote d’Ivoire 2012 (*N* = 3966)		DR Congo 2007 (*N* = 3800)		Ethiopia 2011 (*N* = 13675)	
Characteristic	Odds ratios	95% CI	Odds ratios	95% CI	Odds ratios	95% CI
Age	1.322	(1.217, 1.437)	1.123	(1.030, 1.224)	1.156	(1.061, 1.259)
Age-squared	0.997	(0.995, 0.998)	0.999	(0.997, 1.000)	0.998	(0.997, 1.000)
Urban	3.425	(1.919, 6.111)	1.657	(1.245, 2.205)	2.685	(1.422, 5.069)
Wealth	1.107	(1.040, 1.178)	1.176	(1.117, 1.239)	1.286	(1.127, 1.467)
Wealth*Urban/rural	0.947	(0.867, 1.034)	N/A		0.952	(0.815, 1.113)

Models include: educational level, breastfeeding status, marital status, use of contraception, region of residence, and religion

**Table 3:** Adjusted Odds ratios of being overweight by selected characteristics

	Gabon 2012 (*N* = 4599)		Ghana 2008 (*N* = 4185)		Guinea 2012 (*N* = 3876)	
Characteristic	Odds ratios	95% CI	Odds ratios	95% CI	Odds ratios	95% CI
Age	1.267	(1.169, 1.373)	1.358	(1.259, 1.466)	1.242	(1.140, 1.353)
Age-squared	0.998	(0.996, 0.998)	0.996	(0.995, 0.997)	0.997	(0.996, 0.999)
Urban	2.983	(1.682, 5.290)	3.513	(2.092, 5.900)	2.817	(1.554, 5.106)
Wealth	1.199	(1.083, 1.328)	1.216	(1.138, 1.300)	1.180	(1.076, 1.295)
Wealth*Urban/rural	0.853	(0.758, 0.961)	0.899	(0.826, 0.978)	0.916	(0.826, 1.016)
	Kenya 2008/9 (*N* = 7187)		Lesotho 2009 (*N* = 3593)		Liberia 2007 (*N* = 5757)	
Characteristic	Odds ratios	95% CI	Odds ratios	95% CI	Odds ratios	95% CI
Age	1.216	(1.135, 1.302)	1.188	(1.101, 1.281)	1.277	(1.177, 1.387)
Age-squared	0.998	(0.997, 0.999)	0.998	(0.997, 0.999)	0.997	(0.996, 0.998)
Urban	4.304	(2.466, 7.512)	0.734	(0.424, 1.268)	3.242	(2.299, 4.571)
Wealth	1.239	(1.169, 1.313)	1.150	(1.101, 1.201)	1.348	(1.230, 1.477)
Wealth*Urban/rural	0.824	(0.757, 0.904)	1.043	(0.966, 1.125)	0.790	(0.711, 0.878)
	Madagascar 2009 (*N* = 7153)		Malawi 2010 (*N* = 6405)		Mali 2006 (*N* = 11304)	
Characteristic	Odds ratios	95% CI	Odds ratios	95% CI	Odds ratios	95% CI
Age	1.421	(1.266, 1.594)	1.240	(1.149, 1.336)	1.221	(1.136, 1.312)
Age-squared	0.995	(0.994, 0.997)	0.997	(0.996, 0.998)	0.998	(0.997, 0.999)
Urban	2.255	(1.230, 4.133)	1.800	(1.033, 3.136)	3.907	(2.426, 6.295)
Wealth	1.352	(1.265, 1.444)	1.150	(1.091, 1.213)	1.112	(1.041, 1.189)
Wealth*Urban/rural	0.882	(0.803, 0.969)	0.983	(0.898, 1.076)	0.932	(0.842, 1.032)

Models include: educational level, breastfeeding status, marital status, use of contraception, region of residence, and religion

**Table 4:** Adjusted Odds ratios of being overweight by selected characteristics

	Mozambique 2011 (*N* = 11318)		Namibia 2007 (*N* = 8541)		Niger 2012 (*N* = 3889)	
Characteristic	Odds ratios	95% CI	Odds ratios	95% CI	Odds ratios	95% CI
Age	1.190	(1.127, 1.256)	1.275	(1.202, 1.352)	1.334	(1.216, 1.462)
Age-squared	0.998	(0.997, 0.999)	0.997	(0.997, 0.998)	0.996	(0.995, 0.998)
Urban	1.296	(0.907, 1.853)	1.952	(1.471, 2.589)	3.083	(1.851, 5.135)
Wealth	1.209	(1.152, 1.268)	1.165	(1.125, 1.206)	1.272	(1.177, 1.376)
Wealth*Urban/rural	0.993	(0.938, 1.051)	0.918	(0.881, 0.956)	0.885	(0.798, 0.981)
	Nigeria 2008 (*N*﻿ = 26486)		Rwanda 2011 (*N* = 6178)		Senegal 2010/11(*N* = 4820)	
Characteristic	Odds ratios	95% CI	Odds ratios	95% CI	Odds ratios	95% CI
Age	1.235	(1.197, 1.274)	1.125	(1.060, 1.193)	1.241	(1.155, 1.334)
Age-squared	0.998	(0.997, 0.998)	0.998	(0.997, 0.999)	0.998	(0.997, 0.999)
Urban	1.319	(0.999, 1.740)	1.226	(0.777, 1.938)	2.196	(1.231, 3.917)
Wealth	1.119	(1.093, 1.147)	1.186	(1.128, 1.250)	1.067	(1.017, 1.121)
Wealth*Urban/rural	1.007	(0.969, 1.047)	0.962	(0.898, 1.030)	0.980	(0.907, 1.059)
	Sierra Leone 2008 (*N* = 2960)		Swaziland 2006 (*N* = 4371)		Tanzania 2010 (*N* = 8426)	
Characteristic	Odds ratios	95% CI	Odds ratios	95% CI	Odds ratios	95% CI
Age	1.001	(0.929, 1.089)	1.279	(1.214, 1.347)	1.279	(1.205, 1.358)
Age-squared	1.000	(0.999, 1.001)	0.997	(0.997, 0.998)	0.997	(0.996, 0.998)
Urban	1.715	(0.960, 3.065)	0.994	(0.610, 1.619)	3.129	(2.215, 4.421)
Wealth	0.955	(0.860, 1.060)	1.103	(1.064, 1.145)	1.257	(1.171, 1.350)
Wealth*Urban/rural	1.144	(1.000, 1.308)	0.996	(0.936, 1.060)	0.875	(0.803, 0.953)

Models include: educational level, breastfeeding status, marital status, use of contraception, region of residence, and religion

**Table 5:** Adjusted Odds ratios of being overweight by selected characteristics

	Uganda 2011 (*N* =2201)		Zambia 2007 (*N = *5775)		Zimbabwe 2010 (*N* =7567)	
Characteristic	Odds ratios	95% CI	Odds ratios	95% CI	Odds ratios	95% CI
Age	1.069	(0.950, 1.202)	1.162	(1.078, 1.252)	1.182	(1.122, 1.244)
Age-squared	0.999	(0.998, 1.00)	0.998	(0.997, 0.999)	0.998	(0.998, 0.999)
Urban	2.695	(1.328, 5.471)	3.150	(2.082, 4.765)	1.384	(0.925, 2.071)
Wealth	1.099	(0.966, 1.251)	1.231	(1.165, 1.301)	1.106	(1.066, 1.147)
Wealth*Urban/rural	0.961	(0.828, 1.114)	0.892	(0.832, 0.957)	0.988	(0.933, 1.046)

Models include: educational level, breastfeeding status, marital status, use of contraception, region of residence, and religion

Group (1): the main effects of household wealth and place of residence were statistically significant at 5% level (or of marginal statistical significance) but the interaction of the 2 variables was not statistically significant. The 13 countries in this group were: Burkina Faso, Burundi, Cameroon, Congo Brazzaville, Cote d’Ivoire, Democratic Republic of Congo, Ethiopia, Guinea, Malawi, Mali, Niger, Senegal, and Uganda. In the Democratic Republic of Congo, we did not test the interaction between household wealth and urban/rural residence because there were very few rural households with 4 or more assets. For each level of household wealth, urban women had higher odds of being overweight (on average about 190% higher odds of being overweight than rural women). However, there was wide variation in this association, ranging from 66% higher odds of being overweight among urban versus rural women in DRC [OR = 1.657, 95% CI: 1.245, 2.205], to 515% in Burundi [OR = 6.147, 95% CI: 2.865, 13.188] (see Tables 2, 3, 4, 5).

Group (2): the interaction between household wealth and place of residence was statistically significant and by the principle of parsimony, we treated the main effect of household wealth and urban/rural residence as being statistically significant also regardless of their p-value. The countries in this group were: Botswana, Gabon, Ghana, Kenya, Liberia, Madagascar, Namibia, Tanzania, and Zambia. The interaction effect showed a cross-over pattern where urban women had higher risks of being overweight than rural women at lower levels of household wealth, and rural women had the highest risks of being overweight at higher levels of household wealth. Among women in households with 8 or fewer modern amenities, the mean estimated probability of being overweight for urban women was about 0.28, while that of rural women was 0.21. For wealthier women (in households with nine or more assets), the mean probability of being overweight was 0.46 if a woman lived in an urban area and 0.56 if she lived in a rural area.

Group (3): The associations between overweight status and the place of residence and the interaction effect were not statistically significant at 5% level, while the association with household wealth was statistically significant (except for Sierra Leone). The countries in this group were: Benin, Lesotho, Mozambique, Nigeria, Rwanda, Sierra Leone, Swaziland, and Zimbabwe. For each additional amenity or asset that a household owned, the odds of being overweight increased by between 10% in Swaziland [OR = 1.103, 95% CI: 1.064, 1.145] to 21% in Mozambique [OR = 1.209, 95% CI: 1.152, 1.268].

Descriptive statistics on selected globalization and health indicators according to the 3 groups are shown in Table 6. These show roughly that Group 1 countries had the lowest median per capita GNI ($660) compared with Group 2 ($870) and Group 3 ($800); urbanization rates were lowest in Group 1 (median = 35%), followed by Group 2 (39%) and Group 3 (46%); and the national prevalence of female overweight in 2010 is highest in Group 3 (median = 32%), followed by Group 2 (29%), and least in Group 1 (24%).

**Table 6:** Country groupings and selected macro-level characteristics

GROUP		Per capita Gross National Income, 2012	% population in urban areas, 2012	Life expectancy at birth, 2012	% women 18+ with BMI > = 25 in 2010	% women 18+ with BMI > = 25 in 2014	Adult HIV Prevalence in 2012	Total fertility rate	Country
1	Mean	736	32	59	26	28	2.5	5.7	Burkina Faso, Burundi, Cameroon, Congo Brazzaville, Cote d’Ivoire, Dem Republic of Congo, Ethiopia, Guinea, Malawi, Mali, Niger, Senegal, Uganda
	Median	660	35	58	24	27	1.3	5.7	
	Minimum	240	11	52	17	19	0.5	4.8	
	Maximum	2550	64	64	34	37	10.8	7.6	
2	Mean	2887	43	62	34	38	7.4	4.6	Botswana, Gabon, Ghana, Kenya, Liberia, Madagascar, Namibia, Tanzania, Zambia
	Median	870	39	62	29	33	5.1	4.6	
	Minimum	370	24	57	24	27	0.5	2.7	
	Maximum	10020	86	67	50	53	23.0	6.2	
3	Mean	1465	40	56	33	36	7.3	5.1	Benin, Lesotho, Mozambique, Nigeria, Rwanda, Sierra Leone, Swaziland, Zimbabwe
	Median	800	46	54	32	35	3.1	5.1	
	Minimum	510	19	46	21	24	1.1	3.3	
	Maximum	3100	50	65	46	49	26.5	5.9	

Sources: WHO Global Health Database; World Bank; Demographic and Health Surveys

Gross National Income (Atlas Method, current US$)

Finally, all data were pooled to fit a three-level random effects model to test the third hypothesis. We fitted a sequential model, starting with the individual level variables only and adding the globalization and urbanization variables, and finally the epidemiological variables (see Table 7). The magnitude of the odds ratio for urban/rural residence in the fixed part of the model did not change much with the inclusion of the macro-level variables and remained around 2.1 [95% CI: 2.040, 2.392]. It should be noted that with the exception of the prevalence of female overweight, the macro level variables were not strongly associated with an individual woman’s odds of being overweight. We excluded total fertility rate since this variable had strong correlations with other variables already in the model (GNI and urbanization rate) and its inclusion made the model unstable. We conclude that the third hypothesis was not fully supported by this analysis. However, the 3 groups of countries that emerged in the first stage analysis reflect different patterns of overweight status in rural and urban areas. These groups were broadly explained by levels of national wealth and urbanization, thus suggesting that overweight patterns in rural and urban areas change as countries progress with globalization and urbanization.

**Table 7:** Odds ratios of being overweight using pooled data from 30 African countries

	All Africa (*N* = 208, 656)		Plus globalization variables		Plus health indicators	
Characteristic	Odds ratio	99% CI	Odds ratio	99% CI	Odds ratio	99% CI
Age	1.263	(1.248, 1.278)	1.264	(1.249, 1.279)	1.271	(1.255, 1.286)
Age squared	0.997	(0.997,0.998)	0.997	(0.997, 0.998)	0.997	(0.997, 0.997)
Urban resident	2.112	(1.957, 2.279)	2.133	(1.975, 2.305)	2.209	(2.040, 2.392)
Absolute wealth	1.158	(1.147, 1.169)	1.159	(1.148, 1.170)	1.164	(1.152, 1.175)
Interaction: Wealth*Urban/rural	0.950	(0.938, 0.962)	0.949	(0.937, 0.961)	0.947	(0.935, 0.959)
Macro-level variables						
LN GNI			1.892	(0.910, 3.931)	0.583	(0.303, 1.122)
% urban population			0.994	(0.976, 1.012)	0.991	(0.977, 1.005)
% female overweight					1.041	(1.027, 1.067)
Life expectancy at birth					0.979	(0.949,1.009)
HIV prevalence					0.972	(0.939, 1.006)
Random coefficients						
*Cluster level variance*	*0.218 (0.008)*		*0.231 (0.008)*		*0.264 (0.009)*	
*Country-level variance*	*0.247 (0.064)*		*0.211(0.055)*		*0.069 (0.018)*	

Models include: highest education level, marital status, breastfeeding status, and use of contraception

**Fig. 3:**
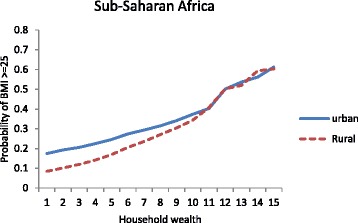
Estimated probabilities of being overweight by urban/rural residence and household wealth for 30 sub-Saharan African countries

For the pooled data, there was a statistically significant interaction between household wealth and place of residence (portrayed by a similar cross-over pattern as that observed for Group 2 countries (see Fig. 3)). This indicates that wealthier women in rural Africa have similar or higher risks of being overweight compared to urban women. Looking at the random components in Table 7, the country-level variation is reduced by 15% when the globalization and urbanization variables are added, and by 72% when the health variables are included. This suggests that much of the variation in the odds of overweight between women in different countries can be explained by the differences in the epidemiological context, particularly the national prevalence of female overweight.

## Discussion

We examined the association between overweight (BMI ≥25) and urban/rural residence and household wealth in sub-Saharan Africa to understand the social and structural determinants of overweight. Like all other regions of the world, the prevalence of overweight is rising in sub-Saharan Africa, and urban living is often cited as the most important structural determinant of this phenomenon. While this was true at the onset of the nutrition transition in SSA, our results show that the transition to higher BMIs is already underway in rural areas and it is happening at low levels of national wealth and urbanization. Previous studies which have investigated the link between urbanization and overweight status in Africa have used simple dichotomies of urban/rural residence without taking into account household wealth, and have thus failed to report the increase in overweight in rural areas. Our results show that wealthier rural women have high risks of overweight similar to urban women and in some countries even higher.

Kennedy et al. and others have linked the rising levels of overweight in LMIC to globalization, urbanization, and socioeconomic drivers including rising household incomes, rural-to-urban migration, and women’s economic empowerment [6, 7]. They argue that these drivers affect food systems (both supply and demand), by altering the production of food (from subsistence to intensive farming), increasing the availability of cheap processed food products because of market liberalization and food imports, changing dietary preferences to ‘western style’ food with high fat and sugar content, and increasing physical inactivity. Very few studies have reported on overweight and obesity among rural women in Africa. A number of studies have documented the changes happening in rural South Africa, where diets high in fat and sugar and sedentary lifestyles have been reported [19, 37, 38]. Only three studies outside South Africa have reported the phenomenon of overweight in rural areas also. Keding et al’s study in rural Tanzania, found that rural women’s diets contained cakes, bread, and high levels of sugar and that this was linked to high BMI [21]. Another study in Mozambique, which was nationally representative, found a narrowing of the gap between affluent urban and rural women in the prevalence of high BMI [39]. That study showed also that the increase in the risk of overweight and obesity occurred with relatively small increases in household income. Another study in eastern Uganda showed that rural and peri-urban areas had quite high levels of overweight women [40]. In terms of changes in levels of physical activity in rural areas of SSA, the evidence is very limited and thought to be unreliable because where validation has occurred, the reliability of such self-reports has been questionable [41].

In our study we identified 3 groups of countries, which can be roughly described as: countries at the start of the nutrition transition (Group 1), where higher household wealth and urban living are associated with high risk of overweight; countries that are rapidly moving towards high levels of overweight (Group 2), where high household wealth and urban living are associated with over-nutrition but sub-groups among rural residents (rural affluent) are over-taking urban residents as being at highest risk of overweight; and countries where female overweight has spread to rural areas so that there is no difference between urban and rural residents (Group 3). There are no studies that report a cross-over pattern in the relationship between overweight and urban/rural residence and socioeconomic status. However, explanations of cross-over patterns in urban settings and changes in food systems provide insights into our findings. Pena and Bacallao discuss the phenomenon of obesity, urbanization and the links with socioeconomic status in Latin America and the Caribbean where the urban poor were found to be particularly at high risk of obesity [42]. They explained the presence of a cross-over pattern in the relationship between obesity and socioeconomic status within urban settings as to do with much higher consumption of fatty and sugary foods among the urban poor than other urban residents. They also noted the high prices of healthier foods such as fruits and vegetables, as has been reported by Hawkes [6]. Ziraba et al. also showed evidence of large *relative* increases between national surveys in levels of obesity and overweight among the poor in urban Africa, and they alluded to the cheapness of high-fat and high-sugar foods relative to healthier options [43].

The results from the pooled analysis show some support for the importance of the epidemiological context, but the association between the odds of overweight and the globalization and urbanization variables that we used was not statistically significant. As SSA countries progress through the development and globalization process, mixed patterns emerge regarding the association between overweight and urban/rural residence. A consistent finding is the strong positive association between household wealth and overweight status, but a weaker association with national wealth which is consistent with other findings [18, 44].

### Study limitations

We highlight some limitations to our study. Firstly, as many other researchers have pointed out, measuring wealth using data from the DHS is imperfect. In this paper we chose to use a proxy for absolute wealth instead of wealth quintiles which are commonly used by many analysts. Although we used absolute wealth index instead of relative wealth quintiles, this choice still does not eliminate the urban bias associated with wealth measures based on assets and amenities found in DHS data. The 2007 BFHS improved on the DHS by collecting additional information on farming equipment, ownership of boats, and other assets that are typically found in rural areas.

A second limitation is that the majority of DHS confine the measurement of anthropometry to young children and women of reproductive ages (15–49 years) so that we do not get a picture from these data of the prevalence of overweight among all adult women and men. Another limitation is that BMI may not be the best indicator of the risk of NCDs as discussed earlier, but currently remains the easiest to measure in household surveys. Finally, the absence of data on the types of food consumed and levels of physical activity limit our full exploration of Kennedy et al’s framework on the relationship between globalization, food systems, and nutritional status.

## Conclusion

This study makes an important contribution to our understanding of patterns of female overweight in both urban and rural sub-Saharan Africa. The paper confirms the hypothesis that household wealth is an important predictor of shifts to overweight status and demonstrates that a simple urban/rural dichotomy is insufficient to understand overweight patterns in Africa. We have shown that in low income and least urbanized countries, urban women have higher risks of being overweight, but that as national wealth and urbanization starts to increase, the association between the place of residence and overweight status is complex. Affluent rural women in such countries are more likely to have higher risks of overweight than urban women. As urbanization approaches 50% and prevalence of overweight increases, the place of residence becomes less relevant compared with household wealth. There is need first and foremost to recognize that the prevalence of overweight women in rural Africa is high and increasing. Studies are needed to understand the shifts in food systems and changes to diets. Policies and programmes are needed to address the high prevalence of overweight status among women to ensure that NCDs do not rise even further.

## Abbreviations

BFHS: Botswana Family Health Survey
BMI: Body Mass Index
DHS: Demographic and Health Survey
GNI: Gross national income
HIV: Human immunodeficiency virus
LMIC: Low and middle income countries
NCDs: Non-communicable diseases
SSA: Sub-Saharan Africa
WHO: World Health Organization
